# Ion recombination corrections of ionization chambers in flattening filter‐free photon radiation

**DOI:** 10.1120/jacmp.v13i5.3758

**Published:** 2012-09-06

**Authors:** Yuenan Wang, Stephen B. Easterling, Joseph Y. Ting

**Affiliations:** ^1^ Melbourne Cancer Center Melbourne FL USA

**Keywords:** flattening filter free, ion recombination, two‐voltage technique, $P_{\text{ion}}$, ionization chamber

## Abstract

The flattening filter free (FFF) X‐rays can provide much higher dose rate at the treatment target compared to the conventional flattened X‐rays. However, the substantial increase of dose rate for FFF beams may affect the ion recombination correction factor, which is required for accurate measurements using ionization chambers in clinical dosimetry. The purpose of this work is to investigate the ion recombination of three types of commonly used ion chambers (Farmer, PinPoint and plane‐parallel) in the FFF photon radiation. Both 6 MV and 10 MV flattened and FFF beams were fully commissioned on a Varian TrueBeam linear accelerator. The ion recombination correction factor, $P_{\text{ion}}$, was determined using the two‐voltage technique for a 0.6 cc Farmer chamber, a 0.015 cc PinPoint chamber, and a 0.02 cc parallel‐plate chamber at different source‐to‐axis distances (SAD) in a solid water phantom or water tank phantom at a depth of 10 cm in a $10\times 10\;{\text{cm}}^{2}$ field. Good repeatability of measurements was demonstrated. Less than 1% difference in Pion between the flattened and FFF photons for all three ion chambers was observed. At a SAD of 100 cm and a depth of 10 cm for a $10\times 10\;{\text{cm}}^{2}$ field, $P_{\text{ion}}$ for the Farmer chamber was 1.004 and 1.008 for the 6 MV flattened and FFF beams, respectively. At the same setup using the Farmer chamber, $P_{\text{ion}}$ was 1.002 and 1.015 for the 10 MV flattened and FFF beams, respectively. All $P_{\text{ion}}$ results for the Farmer, PinPoint, or parallel plate chamber in the 6 MV and 10 MV flattened and FFF beams were within 2% from the unity (1 ≤ $P_{\text{ion}}$ < 1.02). The $P_{\text{ion}}$ ratio of the FFF to flattened beams was 0.99~1.01 for both 6 MV and 10 MV photons. The ion recombination effect of the Farmer, PinPoint, and plane‐parallel chamber in the FFF beams is not substantially different from that in the conventional flattened beams.

PACS number: 87.56.bd

## I. INTRODUCTION

Photon beams from a linear accelerator are traditionally flattened in order to ease dose calculation when computers were not available for treatment planning.^(1)^ However, the conventional flattened beams do not offer advantages for uniform dose distributions because, in reality, the treatment geometry is often curved and tissue inhomogeneity exists. Without the flattening filter in the X‐ray beam path, the radiation output near the central axis and the dose rate at the treatment target have increased significantly,^(2)^ which is especially beneficial to facilitate motion management during stereotactic radiosurgery (SRS) and stereotactic body radiation therapy (SBRT).^3^, ^4^ In addition, the FFF photons provide dosimetric advantages, such as lower head scatter and lower out‐of‐field radiation.^5^, ^6^ For very high photon energies, it has been proposed that fewer neutrons can be produced with the FFF beams and thus unwanted exposure is reduced.^(7)^ With the successful implementation of intensity‐modulated radiation therapy (IMRT) during the last fifteen years and the recent development of volumetric‐modulated arc therapy (VMAT),^8^, ^9^ the need to have a flattened photon beam from a linear accelerator has vanished.

In the absolute dose calibration protocols such as TG‐51^(10)^ or IAEA TRS‐398,^(11)^ the recombination correction factor $P_{\text{ion}}$ for the ion chamber is an important factor to measure. The ionization chamber is the most widely used dosimeter for accurate dose calibrations in radiotherapy quality assurance.^10^, ^11^ For example, a standard 0.6 cc Farmer chamber is commonly used in the radiation beam calibration protocols.^(10)^ A microsized PinPoint chamber can be used to determine relative and absolute dosimetry of small photon fields in the SRS and SBRT procedures.^(12)^ A plane‐parallel chamber, on the other hand, has flat surfaces with thin foils or membranes, which can cause minimal perturbation for incident photons and electrons, and can be also used in radiation beam calibration.^(10)^ Since the Farmer, PinPoint, and parallel‐plate ion chambers are gas‐filled detectors, as charged particles pass through a gas, free electron and positive ion are generated through the ionization process along the track of radiation. By collecting all the charges created in the gas while an electric field is applied, these ion chambers can quantify the radiation dose. However, there is a recombination process when positive ions collide with negative ions^(12)^ to induce charge loss and collection inefficiency. According to Attix,^(13)^ there are two types of recombination: initial and general recombination. The former process occurs most likely in densely ionized track such as α‐particles and is independent of dose rate. The latter process occurs when positive and negative ions from different tracks re‐combine on their way to the collecting electrode and is dose‐rate dependent.

The ion recombination correction factor, $P_{\text{ion}}$, is defined to account for incomplete collection of charges and it is a function of dose per pulse in a linear accelerator.^10^, ^14^ Dose per pulse in the unit of monitor units per pulse (MU/pulse) is dose rate (MU/min) divided by pulse rate (pulse/min). Since pulse rate of the linear accelerator for the same nominal energy does not change, $P_{\text{ion}}$ becomes a function of the dose rate of the photon beams. For the FFF X‐rays, dose rate increases substantially and hence $P_{\text{ion}}$ of the FFF photons may be different from the conventional flattened photons. Therefore, ion recombination in the Farmer, PinPoint, or parallel‐plate ion chambers may vary in the FFF beams. The purpose of this study is to evaluate the ion recombination for typical thimble and plane‐parallel chambers in the FFF photon radiation to facilitate the quality assurance procedure and accurate dose calibrations for the FFF X‐rays.

## II. MATERIALS AND METHODS

Both 6 MV and 10 MV conventional flattened and FFF beams were fully commissioned on a TrueBeam linear accelerator (Varian Medical Systems, Palo Alto, CA). The dose rate was 600, 1400, 600 and 2400 MU/minute for 6 MV flat, 6 MV FFF, 10 MV flat and 10 MV FFF beams, respectively. Three ionization chambers used in the quality assurance procedures for radiation therapy were investigated in this study, which were a standard 0.6 cc Farmer chamber (PTW 30013, Germany), a 0.015 cc PinPoint chamber (PTW 31006, Germany), and a 0.02 cc plane‐parallel chamber (PTW 23342, Germany) (Table 1). A calibrated PC Electrometer (Sun Nuclear Corp, Melbourne, FL) was connected to the ion chamber to measure $P_{\text{ion}}$ in both flattened and FFF beams.

**Table 1:** Specifications of the ionization chambers.

*Chamber Type*	*Manufacturer/Model Number*	*Sensitive Volume (cc)*	*Ion Collection Time (*μ*s)*	*Nominal Response (nC/Gy)*	*Waterproof*
Farmer	PTW 30013	0.6	140	0.2	Yes
PinPoint	PTW 31006	0.015	20	0.4	Yes
Parallel‐plate	PTW 23342	0.02	30	1	No

The two‐voltage technique was used to determine P*ion* for the photon beams^10^, ^15^ with bias high and low voltages of $V_{H}=300\;V$ and $V_{L}=150\;V$. The $P_{\text{ion}}$ was computed using the equation from the TG‐51 protocol:

(1) $$P_{\text{ion}}=(1‐V_{H}/V_{L})/({\text{Mraw}}^{H}/{\text{Mraw}}^{L}‐V_{H}/V_{L})=1/(2‐{\text{Mraw}}^{H}/{\text{Mraw}}^{L})$$

where $\text{Mra}w^{H}$ and $\text{Mra}w^{L}$ were ionization readings at the high and low voltage. Note that it was necessary to wait for chamber readings to reach equilibrium after changing bias voltages.

The $P_{\text{ion}}$ measurement for each ion chamber was conducted at different source‐to‐axis distances (SAD), which were a regular SAD of 100 cm and an extended SAD of 150 cm, to generate different dose rates at the detector. According to the inverse square law, the dose rate ratio was 2.25:1 between the two different SADs. Firstly, the experiments were conducted in a solid water phantom, where each ion chamber was inserted to the phantom at the center of a $10\times 10{\;\text{cm}}^{2}$ field at a depth of 10 cm. All $P_{\text{ion}}$ measurements were conducted at the point of measurement of the ion chamber defined in the TG‐51 protocol.^(10)^ For cylindrical ion chambers such as the Farmer and PinPoint chambers used in this study, the point of measurement was on the central axis of the cavity at the center of the active volume of the cavity. For the plane‐parallel chamber used in this study, the point of measurement was at the front of the air cavity at the center of the collecting region.^(10)^ The cylindrical ion chambers are typically used for photon beam dosimetry in the absolute dose calibration procedure. The parallel‐plate ion chamber, however, is typically used for electron dosimetry. It may also be used in measuring the surface dose or dose in the build‐up region for photon beams in a clinic, where the entrance beam should be perpendicular to the front surface of the parallel‐plate ion chamber. Subsequently, the $P_{\text{ion}}$ measurements for each chamber were compared between the flattened and FFF beam exposures. In addition to the solid water phantom experiments, the $P_{\text{ion}}$ comparison between the FFF and flattened beams for the two water‐proof chambers (Farmer and PinPoint) were conducted in a water phantom at the TG‐51 setup $\left(\text{SSD}=100\;\text{cm},\;\text{FS}=10\times 10\;{\text{cm}}^{2},\;\text{and depth}=10\;\text{cm}\right)$.

## III. RESULTS

**Figure 1:**
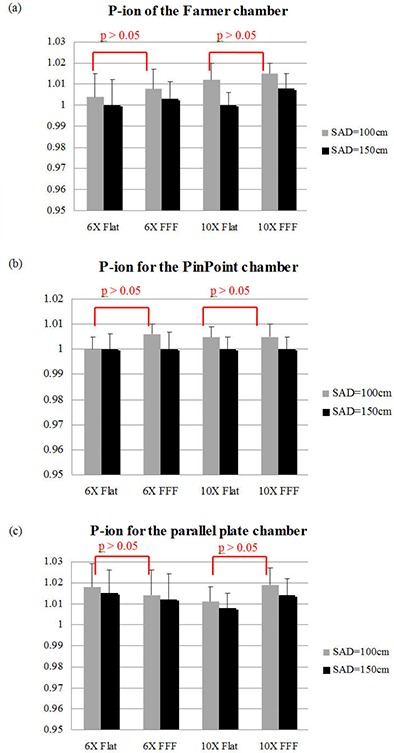
The ion recombination correction factor $P_{\text{ion}}$ of (a) the Farmer chamber, (b) the PinPoint chamber, and (c) the parallel‐plate chamber at two source‐to‐axis distances (SAD). No significant difference in P*ion* between the FFF and flattened beams is observed (error bar=standard deviation; column=mean).

Good repeatability was observed for the $P_{\text{ion}}$ measurements (Fig. 1). The results in Table 2 and Fig. 1 showed that there was less than 1% difference in $P_{\text{ion}}$ of all three chambers at two different SAD between flattened and FFF beams. For instance, $P_{\text{ion}}$ of the Farmer chamber under the 6 MV flattened and FFF beams was 1.004 and 1.008, respectively at the 100 cm SAD. Similarly, the PinPoint chamber and parallel‐plate chamber had less than 1% difference in the ion recombination factor between the flattened and FFF beams. All the average $P_{\text{ion}}$ values in flattened and FFF beam exposures were less than 1.02 for all three types of ion chambers. No $P_{\text{ion}}$ results exceeded 1.05. The $P_{\text{ion}}$ data demonstrated that ion recombination of the three commonly used ion chambers in the FFF beams had very little difference from the flattened beams. For the water phantom test with the TG‐51 setup, the $P_{\text{ion}}$ for the Farmer chamber was 1.005 and 1.009 for the 6 MV flattened and FFF beams, respectively. The $P_{\text{ion}}$ was 1.004 and 1.016 for the 10 MV flattened and FFF beams, respectively. Still the ratio of $P_{\text{ion}}$ of FFF to flattened beams was less than 2%. There was no significantly different $P_{\text{ion}}$ observed between the flattened and FFF photon for both 6X and 10X beams in all three types of ion chambers (Fig. 1).

**Table 2:** The $P_{\text{ion}}$ results of the flattened and FFF beams, where diff (%) = (FFF‐Flat)/Flat.

	$P_{\text{ion}}$					
	*Farmer Chamber*		*PinPoint Chamber*		*Plane‐parallel Chamber*	
SAD (cm)	100	150	100	150	100	150
6X Flat	1.004	1.000	1.000	1.000	1.018	1.015
6X FFF	1.008	1.003	1.006	1.000	1.014	1.012
Diff (%)	0.4%	0.3%	0.6%	0	‐0.4%	‐0.3%
10X Flat	1.012	1.000	1.005	1.000	1.011	1.008
10X FFF	1.015	1.008	1.005	1.000	1.019	1.014
Diff (%)	0.3%	0.8%	0	0	0.8%	0.6%

## IV. DISCUSSION

**Figure 2:**
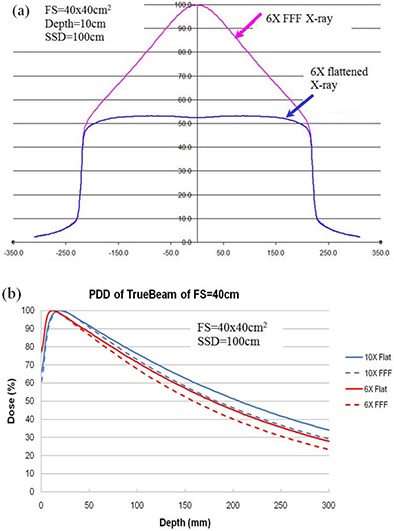
The flattening filter free (FFF) X‐rays (a) have much higher radiation intensity near the central axis than the flattened X‐rays; percent depth‐dose (PDD) curves (b) for 6X and 10X flattened and FFF beams demonstrate that the FFF beams have slightly softer energy spectra than the flattened beams (Varian TrueBeam).

The FFF beams have slightly softer energy spectra than the flattened beam (Fig. 2). There is an increasing interest in the physics and clinical applications of FFF photons recently.^(1–7,16–18)^ The most prominent advantage of FFF beams is the very high dose rate at the treatment target. Since the ion recombination correction required in the quality assurance procedure of clinical dosimetry can be affected by dose rate, the evaluation of ion recombination effect of ion chambers in FFF beams is worthwhile to investigate.

To the best of our knowledge, this work is the first study on the ion recombination corrections of ionization chambers in the FFF X‐ray exposure. Very little difference (i.e., less than 2%) in $P_{\text{ion}}$ has been observed between the FFF and flattened photons at the regular and extended SAD for ion chambers (Table 2). Therefore, similar to the conventional flattened photons, the ion recombination effect of the ion chambers in the FFF photons is insignificant at the operating voltage. Regarding the ion recombination correction, the Farmer, PinPoint, and parallel‐plate ion chambers are suitable to be applied to the quality assurance procedure for FFF beams.

Alfonso et al.^(19)^ have proposed the dosimetry formalism for small and nonstandard fields, where the uncertainty in clinical dosimetry exists due to the lack of charged particle equilibrium. This formalism cannot be directly applied to ion recombination in the ionization chambers; however, $P_{\text{ion}}$ measurement can be also considered as the state of electron unequilibrium. The theoretical approach for estimating recombination corrections has been developed by Boag,^(20)^ and Burns and Rosser.^(21)^ They have shown that simply measuring the charges collected ($Q_{1}$, $Q_{2}$) at two different applied potentials ($P_{1}$, $P_{2}$) can yield an accurate value of collection efficiency for pulsed radiation. The ion collection efficiency for several popular commercial ion chambers has been studied in pulsed and continuous photons using Boag's theory.^(20)^ Weinhouse and Meli^(15)^ provided a convenient method for determining $P_{\text{ion}}$ using the two‐voltage technique with a voltage ratio of two, which was also used in TG‐51 protocol for ion recombination correction. Bruggmoser et al.^(14)^ have proposed that the recombination correction factor depends only on dose per pulse and the chamber type, and it is not affected by radiation type or energy.

One limitation of this study is the use of the two‐voltage method. DeBlois et al.^(22)^ have proposed that the two‐voltage method can result in overestimation of saturation current by 0.7%. They have suggested a semi‐empirical model to obtain more accurate measurement of ion recombination and charge multiplication. Palmans et al.^(23)^ have also proposed that the two‐voltage method is too simple to accurately determine the ion recombination. They have taken the ion chamber geometry into consideration and used Monte Carlo simulation to determine the ion recombination in a helical tomotherapy unit. Their conclusion is that the ion recombination correction can be more accurate by using lower operating voltage rather than the traditional range in the two‐voltage method. Although both studies have realized inaccurate estimation of ion recombination using the two‐voltage method, the two‐voltage method for the $P_{\text{ion}}$ measurement in the TG‐51 practice still can be used in this study because our study is focused on the relative comparison of ion recombination between the FFF and flattened beams. In addition, the two‐voltage method for the $P_{\text{ion}}$ measurement is used as a gold standard in many institutions^10^, ^15^ during X‐ray beam calibration procedures and the ratio of high and low operating voltage bias, which is at least two, can also be used in this study. Nevertheless, the two‐voltage method can introduce systematic error and a more accurate method can be obtained using the models suggested by DeBlois and Palmans.^22^, ^23^

## V. CONCLUSIONS

Although the FFF beams provide much high dose rate at the treatment target, the ion recombination effect of the Farmer, PinPoint, and plane‐parallel chamber in the FFF photons is not significantly different from the flattened photons. These ion chambers are suitable in the quality assurance and exposure measurement for the FFF beams regarding their negligible ion recombination and sufficient collection efficiency.
